# Mechanical circulatory support for haemodynamic protection in patients with heart failure during non-cardiac surgery

**DOI:** 10.1016/j.ahjo.2026.100874

**Published:** 2026-08-27

**Authors:** Tobias J. Pfeffer, Thomas Gausepohl, Vera Garcheva, Bastian Ringe, Anna Koenig, Holger Leitolf, Christoph Terkamp, Constantin von Kaisenberg, Johann Bauersachs, Andreas Schäfer

**Affiliations:** a Department of Cardiology and Angiology, Hannover Medical School, Germany; b Department of General, Visceral and Transplantation Surgery, Hannover Medical School, Hannover, Germany; c Department of Gastroenterology, Hepatology and Endocrinology, Hannover Medical School, Hannover, Germany; d Department of Obstetrics and Gynecology, Hannover Medical School, Hannover, Germany

**Keywords:** Impella, VA-ECMO, Mechanical circulatory support, Heart failure, Non-cardiac surgery

## Abstract

**Background:**

The prevalence of heart failure and the rate of surgical interventions are increasing worldwide, leading to a rising number of patients with heart failure who are at high perioperative risk. We analyzed the value of mechanical circulatory support (MCS) in patients with severe cardiac impairment who underwent non-cardiac surgery.

**Methods:**

Single center retrospective analysis of 5 patients undergoing non-cardiac surgery (NCS) protected by MCS.

**Results:**

Four patients with cardiogenic shock underwent NCS (gland resection, n = 4) protected by veno-arterial extracorporeal membrane oxygenation and an Impella CP microaxial pump. Elective Surgical thyroidectomy under hemodynamic protection with Impella CP was performed in one patient with severe chronic heart failure.

All patients initially survived the surgery, and mechanical circulatory support led to rapid lactate clearance. One patient died during follow-up due to advanced multiorgan failure, the remaining four patients could be successfully weaned from hemodynamic circulatory support. Notably, the patient with fatal outcomes had the longest delay between the onset of cardiogenic shock and initiation of MCS, while other patients with elective or rapid (<24 h) implantation of mechanical circulatory support had favorable outcomes.

**Conclusions:**

MCS is a promising strategy in cardiogenic shock, enabling hemodynamic stabilization and protected surgery. Rapid initiation of MCS is important. This approach may be viable for selected patients with severe heart failure who require elective NCS. Findings are preliminary and hypothesis-generating, further validation is needed.

## Highlights

• Mechanical circulatory support enables hemodynamic stabilization and protected non-cardiac-surgery with a high survival rate.
• Rapid initiation of mechanical circulatory support is important.
• This concept seems to be a promising approach for patients with severe heart failure who require elective non-cardiac surgery.

## Graphical abstract

Mechanical circulatory support during non-cardiac surgery. Created in BioRender. Pfeffer, T. (2025) https://BioRender.com/lug0rc9.

**Figure:**
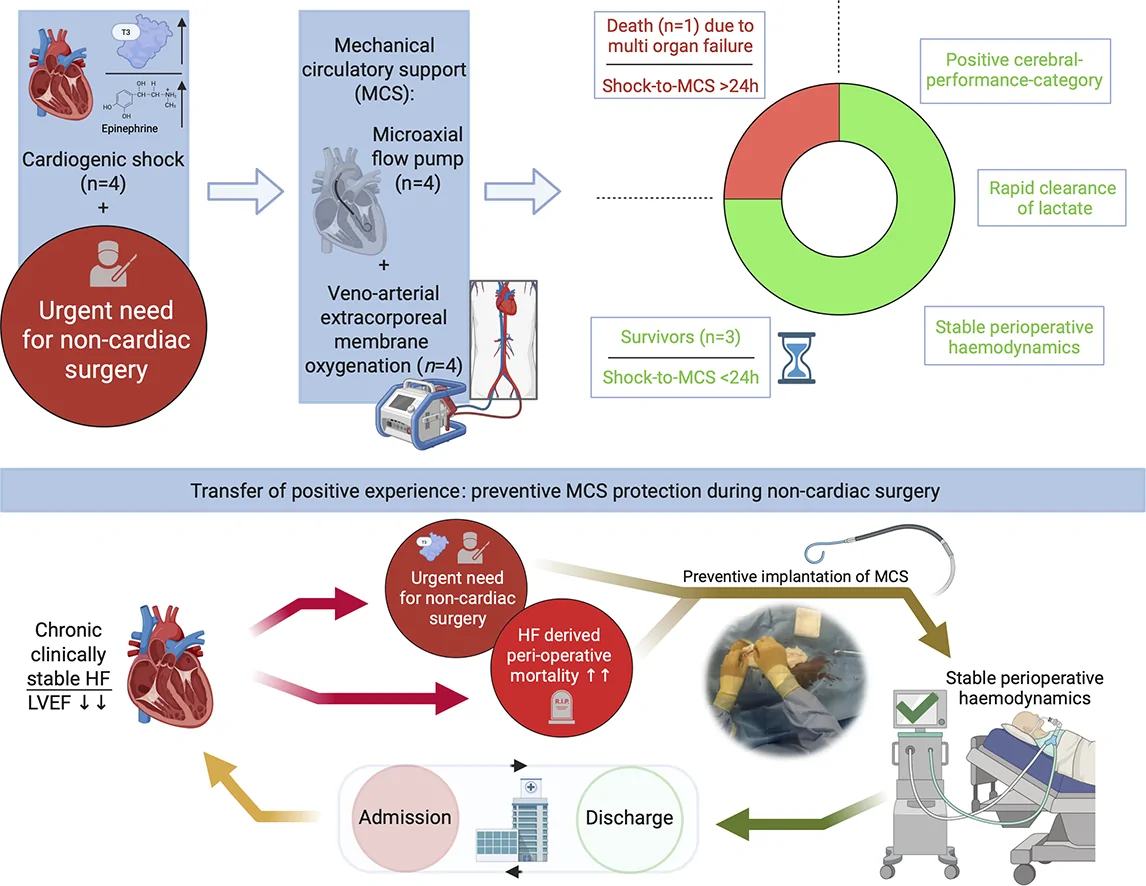

## 1 Introduction

The prevalence of heart failure (HF) is increasing among Western societies due to aging and prolonged survival with chronic HF, owing to advances in pharmaceutic and interventional therapies [1], [2], [3], [4]. Simultaneously, the number of surgical procedures has increased due to demographic changes as well as medical innovations, especially in elderly patients and patients with significant comorbidities [5]. In a large US database of hospital admissions (21,560,996 surgical hospitalizations), heart failure was identified in 4.9% of non-cardiac surgical procedures and was associated with significantly increased risks of perioperative morbidity and mortality [6]. Vice versa triggering pathologies that necessitate surgery can induce worsening or decompensation of HF, and HF itself is associated with an increased rate of comorbidities [7], [8]. In particular, endocrinological disorders often induce acute HF due to hormonal excess, requiring urgent surgical resection of the respective glands in certain cases [9]. Patents with acute HF undergoing major non-cardiac surgery (NCS) displayed an exazerbation of mortality rates (44% vs 11% in those without acute HF at 1 year) [10].

Hence, the number of patients with severe HF and the need for NCS is increasing. Patients with HF are at a higher perioperative risk, which correlates negatively with left ventricular ejection fraction. Notably, this risk is further increased in patients with symptomatic HF [6], [11]. Current clinical guidelines on perioperative cardiovascular management for NCS recommend the continuation of most guideline-directed medical therapy, but discontinuation of sodium-glucose cotransporter-2 inhibitors during elective NCS in patients with HF [12]. Further strategies to reduce cardiovascular risk in patients with HF undergoing NCS are rare [12].

The AHA guidelines on perioperative management for NCS consider the use of mechanical circulatory support (MCS) in patients with severe HF [12]. MCS can be performed using either a percutaneous transvalvular microaxial flow pump (mAFP) and/or venoarterial extracorporeal membrane oxygenation (VA-ECMO). However, mAFP, of which the Impella device family is the most widely used, unloads the failing left ventricle (LV), whereas vaECMO increases LV afterload [13], [14].

In cases of isolated LV failure, MCS may ideally be provided using an mAFP that pumps blood from the LV into the ascending aorta by crossing the aortic valve with a 14F caged device [15]. mAFP therapy may be extended by simultaneous use of VA-ECMO, then named ECMELLA, in cases of worsening hemodynamics or biventricular failure [16]. In principle, mAFP treatment is well established in the clinical setting of cardiogenic shock (CS) [14], [15], [17]. Despite the frequent use of mAFP in CS, data regarding its effectiveness in this scenario were heterogeneous and lacking in randomized controlled trials (RCT) [15], [18], [19]. Recently, the international DanGer Shock trial, a multicenter RCT, demonstrated reduced mortality in patients with acute myocardial infarction (AMI)-related CS by early standardized use of mAFP in addition to standard care compared to standard care alone [20], [21].

Given the positive experience and predictability gained by mAFP in the setting of AMI-CS and other settings, such as mAFP-protected high-risk percutaneous coronary interventions (HRPCI), it is tempting to speculate whether hemodynamic support by an mAFP may be a promising option in patients with severe LV failure and need for NCS [20], [22]. Atoui et al. reported successful hemodynamic support with Impella 2.5 in a patient with severe HF who underwent laparoscopic cholecystectomy for acute cholecystitis [23]. Based on our experience with MCS in other indications and the aforementioned case report, we started to approach MCS-protected NCS in emergencies when MCS was initially implanted for cardiac failure [14], [24], [25], [26]. Furthermore, a patient with severe chronic HF underwent elective MCS-protected NCS based on the expertise gained during emergencies.

The aim of this case series was to report our single-center experience of six patients with severe LV dysfunction when NCS was performed with hemodynamic protection provided by Impella CP alone or in combination with VA-ECMO (ECMELLA).

## 2 Methods

### 2.1 Study design and participants

In this retrospective, single-center case series, we report five cases of patients with severe HF and MCS-protected NCS. Patients who receive Impella implantation in our department are enrolled in the HAnnover Cardiac Unloading REgistry (HACURE), which has a prospective and observational design. The current study was conducted in accordance with the Declaration of Helsinki and was approved by the ethics committee at Hannover Medical School (#3566-2017).

In this case series, we analyzed all patients who met the above-mentioned criteria between 22. January 2022–17. July 2025.

### 2.2 Patient treatment

All patients were transferred to the intensive care unit (ICU) of the Department of Cardiology and Angiology, Hannover Medical School. Criteria for CS were fulfilled when systolic blood pressure was below 90 mmHg and/or inotropes/vasopressors were needed to maintain systolic blood pressure above 90 mmHg and/or signs of end-organ hypoperfusion, such as altered mental status, clammy skin, or elevated lactate (>2 mmol/l) occurred after appropriate correction of preload. Hemodynamic support was established with Impella CP in all four patients. Implantation of the Impella CP was performed via femoral access, and its position was verified every eight hours using transthoracic echocardiography. In cases of concomitant right heart failure defined by central venous pressure above 18 mmHg and cardiac index below 1.5 l/min/m^2^, MCS was extended with implantation of VA-ECMO (ECMELLA) via femoral venous and arterial access [27], [28]. For all patients with ECMELLA, antegrade leg perfusion was maintained using a 6 French femoral catheter to prevent leg ischemia. Anticoagulation during MCS was performed with unfractionated heparin, targeting an activated clotting time of 160–180 s. Patients were treated according to the current European Society of Cardiology guidelines for acute HF and a local standard operating procedure for CS, when applicable [29].

Indications for surgery were made in consultation with the responsible surgeon, and if necessary, an endocrinologist. The prerequisite for surgical treatment under MCS is that omitting the operation poses a greater risk than the surgical risk in the individual patient. In addition, the risk of surgery and anesthesia without MCS due to HF had to be increased to an extent that exceeded the risk due to MCS. The following criteria had to be fulfilled for application of prophylactic MCS: severe left ventricular dysfunction (LVEF <25%), prior MCS support, lack of functional recovery, and urgent need for non-cardiac surgery due to life-threatening hormone excess.

### 2.3 Statistical analysis

Numbers are presented as n (%) and mean ± standard deviation (SD) for normally distributed variables, or median and interquartile range (IQR) for non-normally distributed variables in the tables. Data analysis was performed using GraphPad Prism 10.0 (GraphPad Software, Inc., La Jolla, California, USA).

## 3 Results

### 3.1 Patient characteristics

Between January 2022 and July 2025, MCS protected NCS was performed in five patients with a median age of 53 years. In four of these patients, MCS was used in CS for hemodynamic stabilization and to allow emergency surgery (Table 1, Table 2), whereas it was used in a purely elective approach solely for patient 2. This patient was the only patient with pre-existing HF, whereas the other previously heart-healthy patients required MCS due to fulminant endocrinological diseases, as reported in Suppl. Table 1. Two patients were transferred from the referring hospitals, whereas three patients were initially treated at the Hannover Medical School.

**Table 1:** Baseline characteristics.

Baseline characteristics	Median (IQR), n/total (%); n = 6
Age (years)	54 (46–59)
BMI (kg/m^2^)	25 (24–32)
Height (cm)	169 (163–180)
Weight (kg)	90 (72–113)
Smoking	1/5 (20%)
Chronic obstructive pulmonary disease	0/5 (0%)
Arterial hypertension	3/5 (60%)
Diabetes mellitus	2/5 (40%)
Pre-existing heart failure	1/5 (20%)
Severe reduced LVEF	5/5 (100%)
Chronic renal failure	1/5 (20%)
Liver cirrhosis	1/5 (20%)
Acute respiratory distress syndrome	1/5 (20%)
Transferred from secondary hospital	2/5 (40%)
V-A ECMO in cardiac arrest (ECPR)	2/5 (40%)

Numbers are presented as median with interquartile range (IQR) or numbers (n/total) and percentage (%) respectively. BMI – Body-mass index, ECPR - Extracorporeal Cardiopulmonary Resuscitation, V-A ECMO – veno-arterial extracorporeal membrane oxygenation.

**Table 2:** Intensive care treatment and mechanical circulatory support.

Intensive care treatment	Median (IQR), n/total (%); n = 6
Duration intensive care unit treatment (days)	26 (5–32)
Impella CP	5/5 (100%)
Veno-arterial extracorporeal membrane oxygenation (ECMO)	4/5 (80%)
Cardiopulmonary resuscitation	2/5 (40%)
SCAI shock stage A/B/C/D/E	1/0/0/1/3
Tachycardic arrhythmia	3/5 (60%)

**Shock to Impella-insertion-time (n** **=** **5)**	
<6 (h)	2/4 (50%)
6–12 (h)	0/4 (0%)
12–24 (h)	1/4 (25%)
>24 (h)	1/4 (25%)
Duration of Impella support (h)	220 (50–356)
ECMO primary to Impella	4/4 (100%) (n = 4)
Duration of Shock to ECMO (h)	2 (1–37)
Duration of ECMO support (h)	226 (97–359)
Duration of combined support (h)	224 (95–341)
Duration Impella to surgery (d)	1 (1–6)
Emergency surgery	4/5 (80%)
Mechanical ventilation (except during surgery)	4/5 (80%)

**Complications during MCS**	
Haemolysis	0/5 (0%)
Acute kidney injury	3/5 (60%)
Thrombolysis in Myocardial Infarction bleeding Score	
None	1/5 (20%)
Minimal	0/5 (0%)
Minor	2/5 (40%)
Major	2/5 (40%)
Bleeding site	
Operation site	1/5 (20%)
Cannulation site	4/5 (80%)
Oral	2/5 (40%)

**Time of bleeding (n** **=** **4)**	
During MCS	4/4 (100%)
Before MCS	0/4 (0%)

**Medical treatment**	
Levosimendan	3/5 (60%)
Norepinephrine	5/5 (100%)
Epinephrine	1/5 (20%)
Dobutamine	3/5 (60%)
Vasopressin	1/5 (20%)

Numbers are presented as median with interquartile range (IQR) or numbers (n/total) and percentage (%) respectively. ECMO - extracorporeal membrane oxygenation; MCS – mechanical circulatory support; SCAI - Society for Cardiovascular Angiography & Interventions.

### 3.2 Mechanical circulatory support and intensive care treatment

Impella CP was used in all five patients and was accompanied by VA-ECMO in four patients presenting as emergency cases requiring urgent surgery, mostly because CS was attributable to endocrinological disorders, leading to hormone excess. The median duration of ICU stay was 29 days (range: 3–32 days). One patient died despite MCS and surgery because of advanced multiorgan failure (Table 2).

**Fig. 1:**
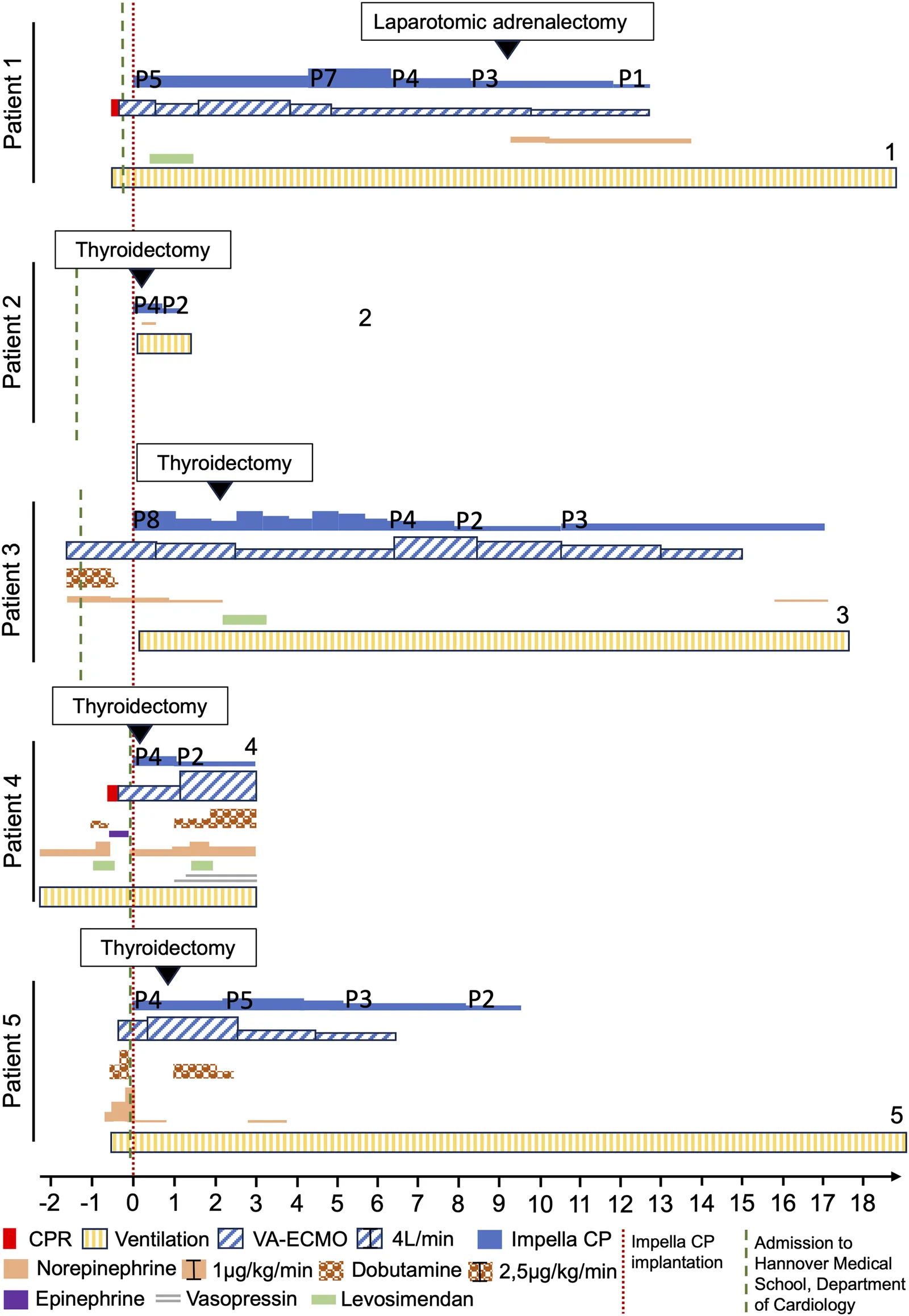
Time course of treatment for patients protected with mechanical circulatory support (MCS) during non-cardiac surgery presented as mean ± standard deviation. CPR: cardiopulmonary resuscitation; VA-ECMO: veno-arterial extracorporeal membrane oxygenation. 1 = d1–18 mandatory ventilation, d18–32 assisted ventilation. Then transfer and successfull weaning in external weaning clinic, 2 = Demission, 3 = d2–22 mandatory ventilation including weaning (succesfull), 4 = death, 5 = d1–32 mandatory ventilation, then transfer to external weaning clinic.

The detailed treatment of all five patients is shown in Fig. 1. Of note, patient 1 had refractory CS with pulseless electrical activity in an external hospital; therefore, transfer to our cardiac arrest center was performed with ongoing CPR using the Lund University Cardiac Assist System (LUCAS) Chest Compression System, VA-ECMO was immediately established after arrival. Patient 3 also suffered refractory CS due to thyrotoxic crisis after presentation at an external hospital. Hence, VA-ECMO implantation was performed on-site after deploying an ECMO team prior to patient transfer. Patient 4 was treated in another department at our hospital when developing asystole due to thyrotoxic crisis; therefore, the VA-ECMO system was installed during resuscitation.

As stated in Table 2, all patients receiving ECMELLA were supplied with VA-ECMO prior to Impella implantation, with a median time from diagnosis of CS to VA-ECMO implantation of 2 h. Ventricular unloading by Impella CP was performed within the first 24 h in three patients with CS. As shown in Fig. 1 and Table 2, MCS was established with a profound delay (after 48 h) in patient 4. The median duration of VA-ECMO support was 226 h, and the median duration of Impella support was 220 h. Combined MCS was performed in a median of 224 h, with a large range of 95–341 h. The time on MCS was by far the shortest in patient 2, who presented without CS and in whom Impella could be explanted within 24 h after elective implantation and surgery (Fig. 1).

### 3.3 Catecholamines and haemodynamics

**Fig. 2:**
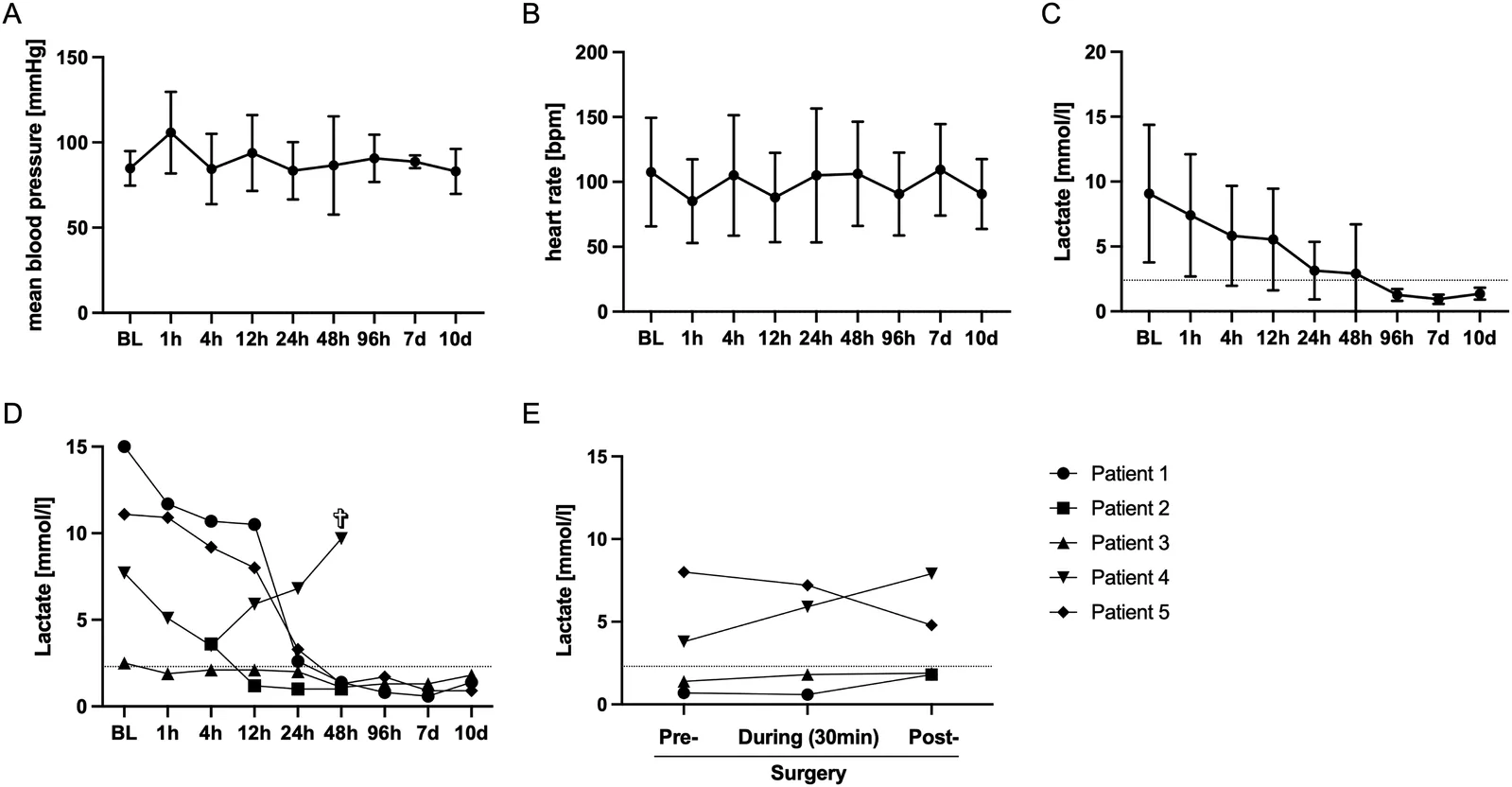
Mean blood pressure (A), heart rate (B) and serum lactate levels (C, D) after left ventricular unloading by Impella implantation (Baseline) presented as mean ± SD (A–C). Serum lactate levels prior, during and after non-cardiac surgery (E). BL = Baseline, ✞ = death.

The variety and levels of catecholamines were higher in patients who underwent emergency surgery (Tbl.1+, Fig. 1). mAFP implantation allowed for rapid weaning of catecholamines in patients with CS (except in patient 1, who initially did not require vasopressors owing to endogenous catecholamine excess derived from pheochromocytoma). Blood pressure and heart rate remained stable after catecholamine reduction under MCS (Figs. 1 & 2 A & B).

In addition, there was a gradual clearance of lactate to the normal range within the first 48 h after Impella implantation, with the exception of patient 4 (Fig. 2 C&D). After an initial decline in catecholamine requirements and lactatemia in patient 4, the patient developed multiorgan failure with progressive lactate release and significant catecholamine and vasopressor demands.

### 3.4 Surgery

The duration between the first medical contact and surgery ranged from one to ten days. All five patients received surgical removal of a hormone excess-inducing gland (4× thyroid, 1× adrenal gland) under general anesthesia and MCS protection. In four of these patients, hormonal excess was the trigger for cardiac dysfunction. Further details regarding indications and surgery are presented in Suppl. Table 1.

### 3.5 Outcome

All five patients survived the MCS-protected surgery (Fig. 2 E). However, patient 4 died two days thereafter. The other four patients were successfully weaned from MCS and survived, as shown in Suppl. Table 2. The complications during MCS are presented in Table 2. Major bleeding under MCS, classified by the Thrombolysis in Myocardial Infarction (TIMI) bleeding score, occurred in two patients. One patient had major bleeding at the insertion sites for the cannulas of the MCS devices, while patient 1 was bleeding in the resection area after surgery. All five surviving patients showed a positive neurological outcome, as indicated by a Cerebral Performance Category (CPC) Score of one or two.

## 4 Discussion

In this study, we present our findings on percutaneous MCS using the mAFP Impella CP in one case of severe LV dysfunction and elective NCS and the combination of Impella CP and VA-ECMO for biventricular failure in four patients with CS and the need for emergent NCS. MCS-protected NCS have previously been reported only in single case reports. Furthermore, temporary MCS protection is a valuable tool for HF patients undergoing bariatric surgery [23], [30], [31]. In contrast, MCS during cardiac surgery is well established and widely recognized as an effective strategy for high-risk patients, with strong recommendations and detailed guidance provided in recent consensus statements and guidelines from major professional societies [32], [33]. To the best of our knowledge, however, this is the largest series on the topic, including one innovative case of elective MCS-protected NCS.

The mortality rate of CS due to hormone excess is high, and in thyrotoxicosis, the estimated HF rate is approximately 30% [34], [35]. Four patients had biventricular CS due to hormone excess, and pharmacological therapy to stabilize hemodynamics was insufficient or pathophysiologically detrimental (e.g., catecholamine therapy in pheochromocytoma) [36]. MCS initially ensured survival in these patients by stabilizing hemodynamics, which is consistent with reports of the beneficial effects of MCS in the setting of CS during thyrotoxicosis [34]. In addition, MCS enables surgical gland resection and is a key feature in overcoming the dilemma of an urgently needed resection of the CS-causing gland in the context of immense high perioperative risks due to CS [6], [11], [35]. Owing to its ability to immediately compensate for declining cardiac output and the benefits derived from ventricular unloading, mAFP implantation as hemodynamic support was established during coronary interventions with a high periprocedural risk [15]. Studies on the use of mAFP during HRPCI or ablation of ventricular tachycardia in selected high-risk patients showed heterogeneous results [22], [37]. The properly designed RCT investigating MCS-protected HRPCI with mAFP was prematurely terminated due to presumed futility; however, upon analysis of the trial, there appeared to be a non-significant benefit of the predefined primary endpoint, which became significant upon extended follow-up [22].

Patient 4 had the longest duration (48 h) from onset of CS to initiation of MCS. This patient died despite successful MCS-protected thyroidectomy due to advanced multiorgan failure of mixed etiology, characterized by refractory cardiogenic shock, acute liver failure and acute kidney injury with features suggestive of septic pathogenesis. This highlights the importance of rapid implementation of MCS and matches with experiences in the context of AMI-CS or refractory CS in PPCM, where early MCS implementation had beneficial effects on survival and/or outcome when implemented prior to potential multiorgan dysfunction [24], [38]. Furthermore, MCS enabled rapid reduction of dobutamine and norepinephrine use, as shown in patients 3 and 5 (Fig. 1).

VA-ECMO increases LV afterload via retrograde blood flow, leading to a high risk of pulmonary congestion and LV-thrombus formation [39]. With regard to the high 30 day survival of 75% among patients with CS, LV unloading using Impella CP proved to be effective in stabilizing hemodynamics and organ perfusion, as indicated by the rapid reduction in lactate levels of −72% in the first 24 h (Fig. 2 C&D) [40]. Ventricular unloading was performed with Impella CP in all four patients receiving VA-ECMO. Reduction of myocardial oxygen consumption due to decongestion by Impella CP seems to be an important benefit in the clinical context of hormonal excess and NCS/anesthesia, as catecholaminergic and thyrotoxic crises per se enhance myocardial oxygen turnover [34], [41]. Second, all patients underwent surgery and anesthesia, which is known to reduce myocardial oxygen utilization efficiency [42]. Investigation of the effects of mAFPs in large animal models identified reduction in ventricular pressure, consecutively reduced myocardial oxygen consumption, and elevated cardioprotective signalling as its beneficial features [43], [44].

Bleeding was the most common complication during MCS and major bleeding occurred in 40% of the patients in this study as shown in Table 2. Although our cohort is much smaller, bleeding seemed to be more frequent than in studies with a different MCS indication, such as AMI-CS (DanGer-Shock study: 26,3%) [20]. The high risk of perioperative bleeding is most likely due to the combination of surgery on heavily perfused glands and intensive anticoagulation required for MCS [20]. Therefore, intraoperative hemostasis and careful postoperative bleeding monitoring are of great importance during MCS-protected surgery.

In patient 2, resection of the thyroid gland was indicated, but there was a high intraoperative risk due to severe cardiac failure after AMI-CS (Tabl. 3). Anesthesia affects the sensitive balance of sympathetic tone in patients with HF, who are therefore at a higher risk of periprocedural decompensation due to peripheral vasodilatation and myocardial depression [45]. Hence, we targeted the risk of myocardial depression in patient 2 by preemptive use of mAFP-derived MCS, enabling the adaptation of cardiac output and unloading of the LV during anesthesia and NCS. The duration of perioperative MCS was short, as is known from other mAFP-supported procedures, such as HRPCI, and acute cardiac decompensation was prevented [22].

### 4.1 Conclusion

In conclusion, our study suggests that MCS may enable safe performance of NCS in the context of severe HF and CS, particularly in patients with high risk of intraoperative cardiac decompensation. The findings are preliminary and should be interpreted as hypothesis-generating rather than definitive. In CS in particular, rapid establishment of MCS appears to be relevant to prognosis. While elective MCS-protected NCS shows potential benefit in selected high-risk patients, these results require validation in larger, prospective studies.

### 4.2 Limitations

Several limitations must be mentioned. This observational series showed a modest number of patients with MCS protected NCS. The patient collective was very heterogeneous regarding the underlying cause of HF and the permormed NCS, and included emergent and non-emergent cases. Furthermore, no control group was included. As the presented patient collective is rare but growing, this study may provide hypotheses and may help to initiate larger and controlled trials to generate data on the effectiveness of MCS protected NCS.

## CRediT authorship contribution statement

**Tobias J. Pfeffer:** Writing – review & editing, Writing – original draft, Visualization, Validation, Software, Investigation, Formal analysis, Data curation, Conceptualization. **Thomas Gausepohl:** Writing – original draft, Investigation, Formal analysis, Data curation. **Vera Garcheva:** Writing – review & editing, Investigation, Data curation. **Bastian Ringe:** Writing – review & editing, Investigation, Data curation. **Anna Koenig:** Writing – review & editing, Investigation, Data curation. **Holger Leitolf:** Writing – review & editing, Investigation, Data curation. **Christoph Terkamp:** Writing – review & editing, Investigation, Data curation. **Constantin von Kaisenberg:** Writing – review & editing, Investigation, Data curation. **Johann Bauersachs:** Writing – review & editing, Writing – original draft, Validation, Supervision, Resources, Project administration, Funding acquisition, Conceptualization. **Andreas Schäfer:** Writing – review & editing, Writing – original draft, Visualization, Validation, Supervision, Software, Resources, Project administration, Methodology, Funding acquisition, Formal analysis, Conceptualization.

## Ethical statement

The current study was conducted in accordance with the Declaration of Helsinki and was approved by the ethics committee at Hannover Medical School (#3566-2017).

## Funding

The HACURE registry is funded by an institutional grant from Abiomed.

## Declaration of competing interest

AS received lecture fees and honoraria from Amgen, AOP, Boehringer Ingelheim, Daiichi-Sankyo, Eli Lilly, Johnson&Johnson MedTech, Novartis, Pfizer, ZOLL as well as research support by Johnson&Johnson MedTech and Daiichi-Sankyo. JB received lecture fees and honoraria from Novartis, Abbott, Bayer, Pfizer, Boehringer Ingelheim, AstraZeneca, Eli Lilly, Cardior, CVRx, BMS, Amgen, Edwards, NovoNordisk, Roche, ZOLL not related to this article; and research support for the department from ZOLL, CVRx, Johnson&Johnson MedTech, Norgine, Roche, not related to this article.

TJP received honoraria for lectures/consulting from AstraZeneca and AOP Health, not related to this article.

The other authors declare that they have no conflicts of interest, particularly not related to the current manuscript.
