# Supplementary material for: Mechanical circulatory support for haemodynamic protection in patients with heart failure during non-cardiac surgery
Source: Am Heart J Plus. 2026 Aug 27;70:100874. doi: 10.1016/j.ahjo.2026.100874 (PMC13571505; doi:10.1016/j.ahjo.2026.100874)
Supplement: Supplementary file 1 [file mmc1.docx]

| Patient number | Anesthesiologic procedure: medication (dose) | Duration of surgery (min) | Surgery | Duration initial medical contact /surgery (d) | Duration initial medical contact/admission ICU, MHH Cardiology (d) | Event/Indication for MCS protected noncardiac surgery |
| --- | --- | --- | --- | --- | --- | --- |
| 1 | Sufentanyl (225µg),  Propofol (100mg)  Midazolam (10,6mg),  Sevoflurane  Rocuroniumbromide (80mg) | 93 | Laporotomic adrenalectomy | 10 | Same day transfer | CS by catecholaminergic crisis/ruptured pheochromocytoma right adrenal gland |
| 2 | Remifentanyl (6188µg)  Propofol (478mg)  Sevoflurane  Rocuroniumbromide (40mg) | 134 | Thyroidectomy | (n.a./  elective) | (n.a./  elective) | Amiodarone-induced hyperthyroidism / severe heart failure post AMI-CS (LVEF 22%) |
| 3 | Sufentanyl (90µg)  Propofol (1440 mg) Rocuroniumbromide (40mg) | 117 | Thyroidectomy (+partial parathyroidectomy/ autotransplantation) | 4 | Same day transfer | CS by thyrotoxic crisis  (Grave's disease) |
| 4 | Sufentanyl (208µg)  Midazolam (7,8mg)  Sevoflurane  Rocuroniumbromide (50mg) | 120 | Thyroidectomy (+open implantation of cannula for distal leg perfusion for VA-ECMO) | 4 | 4 | CS by thyrotoxic crisis  (Grave's disease + amiodarone treatment for atrial fibrillation) |
| 5 | Sufentanyl (241µg)  Midazolam (9,1mg)  Propofol (1338mg) Rocuroniumbromide (100mg) | 105 | Thyroidectomy | 1 | Same day transfer | CS by thyrotoxicosis  (Grave's disease + iodine exposure by contrast medium administration) |

**Supplemental Table 1:**

Supplemental Table 1: Indication and characteristics of Surgery

AMI-CS=Acute Myocardial Infarction complicated by Cardiogenic Shock (CS); ECMO - extracorporeal membrane oxygenation; ICU – intensive care unit; LVEF – left-ventricular ejection fraction; MCS – mechanical circulatory support.

**Supplemental Table 2:**

| Clinical endpoints | *n*/total (%); *n*=6 |
| --- | --- |
| Survival surgery | 5/5 (100%) |
| 30 days survival | 4/5 (80%) |
| Successful weaning from MCS | 4/5 (80%) |
| Cerebral performance category Score at discharge | |
| CPC 1 | 3/5 (60%) |
| CPC 2 | 1/5 (20%) |
| CPC 3 | 0/5 (0%) |
| CPC 4 | 0/5 (0%) |
| CPC 5 | 1/5 (20%) |

Supplemental Table 2: Surgical- cardiac- and neurological outcome

Numbers are presented as numbers (n/total) and percentage (%) respectively.

CPC - Cerebral Performance Category; MCS – mechanical circulatory support.
